# Clinical Success Evaluation of Ultrathin Ceramic Veneers Bonded to Nonprepared Teeth: An Observational Prospective Cohort Study

**DOI:** 10.7759/cureus.68699

**Published:** 2024-09-05

**Authors:** Abdulhamid Al-Mokdad, Eyad Swed, Mohanad Kadhim, Shaza Kanout, Ahmad Al-Mokdad, Anas Abdo, Mohammad Y. Hajeer

**Affiliations:** 1 Department of Fixed Prosthodontics, Faculty of Dentistry, University of Damascus, Damascus, SYR; 2 Department of Fixed Orthodontics, Faculty of Dentistry, University of Damascus, Damascus, SYR; 3 Department of Periodontology, Faculty of Dentistry, Ibn Hayyan University, Najaf, IRQ; 4 Department of Fixed Prosthodontics, Faculty of Dentistry, Syrian Private University, Damascus, SYR; 5 Department of Orthodontics, Faculty of Dentistry, University of Damascus, Damascus, SYR

**Keywords:** ultrathin ceramic veneers, failure rate, success rate, ceramic veneers, mechanical properties, tooth preparation, lithium disilicate glass ceramic

## Abstract

Background: Patients' increasing interest in achieving optimal cosmetic outcomes and the widespread use of ultrathin ceramic veneers offer advantages such as high esthetic results and long-term durability. Several issues related to tooth preparation have been raised, including dental sensitivity, periodontal diseases, and increased treatment phases, in addition to complications associated with previous procedures, the treatment of which remains controversial to date. With the advancement of dental ceramic and its manufacturing techniques, it was widely used to manufacture ultrathin ceramic veneers with minimal preparation. Issues such as fracture and abfraction are the most common in ceramic veneers made of feldspathic ceramic due to their weak mechanical properties against various forces, which led to the emergence of lithium disilicate glass-ceramic manufactured using the heat-press technique. This has resulted in ultrathin ceramic veneers with a thickness of up to 0.1-0.2 mm easily bonded and finished as they have high mechanical properties and esthetic qualities that mimic natural tooth color and shape. The current cohort study aimed to evaluate the success rates of this kind of treatment for patients treated at our department.

Materials and methods: This observational cohort study's sample comprised 60 ultrathin ceramic veneers manufactured from lithium disilicate glass-ceramic bonded to nonprepared upper teeth. The clinical performance of the studied sample was evaluated and monitored at monthly intervals (one month, three months, six months, and one year) using the clinical success evaluation based on Walton's principles adopted for evaluating the success and failure of fixed restorations.

Results: Ultrathin ceramic veneers made from lithium disilicate glass-ceramic, bonded to nonprepared teeth, proved to be a successful clinical and esthetic treatment option, with a clinical success rate of 100% during the entire follow-up period.

Conclusions: This study's findings indicate that ultrathin ceramic veneers made from lithium disilicate glass-ceramic, bonded to nonprepared teeth, are a successful clinical and esthetic treatment option, with a clinical success rate of 100% during the entire follow-up period.

## Introduction

The term "clinical success of ceramic veneers" refers to the absence of the need for restorative retreatment, indicating that the restoration is successful in all vital, functional, and esthetic aspects [1]. The term "clinical survival" refers to the presence of the restoration with the possibility of partial failure requiring intervention without absolute failure [2]. In recent years, traditional concepts for ceramic veneer preparation have evolved, which previously required dental tissue preparation to ensure sufficient space for manufacturing veneers approximately 1 mm thick at the cervical margin and 0.5 mm at the finish line, depending on the type of horizontal preparation [3]. Horizontal preparation types include shoulder, chamfer, and simple finish line, with shoulder preparations providing higher fracture [4].

Despite the advantages of a simple finish line for matching the nature of the boundary between the enamel and cementum, reducing the thickness of the enamel in the cervical area maintains enamel tissues [5]. In recent years, vertical preparation for ceramic veneers without a finish line has emerged. This technique is considered more conservative for dental tissues and ensures marginal adaptation [6]. Studies prefer vertical preparation over traditional preparation for periodontal and gingival health, improving the emergence profile of restorations [7]. Determining the distance between the contact point of nonprepared ceramic veneers and the crest of the alveolar bone is crucial, especially in cases of interdental spaces, as this affects the interdental papilla [8]. When this distance is normal, the papilla fills the entire gingival space, but when the distance is 6 mm or more, black triangles are formed in 63% of cases [9].

Ultrathin ceramic veneers are similar to traditional veneers, but they require minimal or no preparation [10], such as removing fixation areas hindering veneer adaptation or rounding sharp or rough angles in which occlusal forces are concentrated, leading to veneer fractures [11]. Nonprepared ultrathin ceramic veneers made of feldspathic ceramic are fragile due to their low mechanical properties, with a flexural modulus of 62-90 MPa, prone to fracture due to the presence of a glass matrix and absence of filler materials [12]. Morimoto et al.'s systematic review found a cumulative survival rate of feldspathic ceramic veneers with traditional preparation at 87%, with less strong attachment to exposed dentin, which may harm the pulp during etching with phosphoric acid [13]. Conversely, Smielak et al. concluded that the clinical survival rate of feldspathic ceramic veneers with minimal preparation exceeds that of traditionally prepared veneers due to their high bonding strengths and proper adhesion to intact enamel, reducing marginal leakage and discoloration [1].

The traditional cosmetic veneer technique in the medical literature is based on tooth preparation [14]. Ceramic veneers with a thickness sufficient to obtain the desired result have been made of feldspathic ceramic using the refractory die technique or platinum foil [15]. Then, they were made of lithium disilicate glass-ceramic using the heat press or computer-aided design/computer-aided manufacturing technique. These veneers mimic the esthetic properties of feldspathic ceramic veneers while surpassing them in mechanical properties, thus eliminating the issues associated with feldspathic ceramic veneers, such as fractures and abfractions, while preserving the integrity of the healthy dental tissues.

Regardless of the ceramic used and the manufacturing method, preparing teeth with thicknesses of 0.3-0.7 mm to receive the veneers involves many negative aspects that affect their durability, such as tooth sensitivity, discoloration, pulp necrosis, and debonding of the veneers as a result of adhering them to the exposed dentin during preparation, which is considered an irreversible process [1]. Commercial types of ultrathin ceramic veneers are available, such as Lumineers produced by Den-Mat in California, which are ceramic veneers made from Cerinate, a glass ceramic reinforced with leucite, characterized by low viscosity, allowing very thin application [16]. As a result of the development of dental ceramic systems and the modern techniques used to manufacture ceramic veneers, impression materials, and resin cement [17], the increased importance of preserving healthy enamel structure and thus no or minimal preparation of the teeth came back to the surface after the condition had been well diagnosed. Making no-prep or minimal-prep cosmetic veneers is sometimes an alternative to traditionally prepared veneers, which requires removing large amounts of healthy enamel [18].

Fractures and abfractions are the most common complications of ultrathin ceramic veneers, and they have been the subject of research in recent years [10,19]. Many laboratory and clinical research and retrospective clinical studies have been published that have considered minimal-prep ceramic veneers to be an acceptable conservative treatment option that preserves dental structures and is capable of changing the color and shape of teeth in cases of diastema, peg-shaped incisors, tooth discoloration, and others [20,21]. However, recent clinical studies that used feldspathic ceramic to make no-prep veneers replaced the failed veneers with ceramic veneers made of lithium disilicate glass-ceramic without much preparation to preserve dental tissues. Gonzalez-Martin et al. replaced 20% of the failed feldspathic ceramic veneers with veneers made of lithium disilicate glass-ceramic [10]. Mihali et al. replaced all failed ceramic veneers with veneers made of lithium disilicate glass-ceramic [12]. Despite the widespread use of ultrathin ceramic veneers made of lithium disilicate glass-ceramic, no studies have evaluated this treatment modality when used on nonprepared teeth. Therefore, the current cohort study aimed to evaluate the clinical success of these veneers in our department.

## Materials and methods

Study design and settings

The study was an observational prospective cohort study using ultrathin ceramic veneers on vital nonprepared upper teeth. The treatment was provided at the Department of Fixed Orthodontics, Faculty of Dentistry, University of Damascus, Syria. The study was approved by the Local Research Ethics Committee of the University of Damascus (UDDS 332419052022/SRC-278). The University of Damascus funded the study (reference no.: 501100020595).

Patient recruitment and inclusion criteria

The clinical sample consisted of patients referred to the Fixed Prosthodontics Department at the Faculty of Dentistry, University of Damascus. Sixty ultrathin ceramic veneers made from lithium disilicate glass ceramics were fixed to their upper teeth without abutment preparation.

The inclusion criteria were: (1) patients aged over 18 years and less than 45 years, (2) good oral health, (3) vital upper teeth that would serve as abutments, (4) healthy periodontal tissues, (5) absence of temporomandibular joint disorders, and (6) absence of any evident malocclusion. Patients who agreed to participate were asked to sign the related consent form and the observation during the follow-up period.

Clinical procedures

Each patient was examined, and the general and oral history was obtained using a specific form. Radiographs were taken for abutment examination and general dental conditions. Photographic records were obtained, and initial impressions were taken using condensation silicone (Zetaplus, Zhermack, Badia Polesine, Italy) to study and document the case on gypsum models (Apex RZ, Garreco Dental, Heber Springs, AR).

Periodontal treatment commenced with tooth cleaning and scaling, emphasizing oral care instructions. Cases were studied using Exocad software (DentalCAD®, Darmstadt, Germany), and various treatment options were explained to the patient. Written consent was obtained after discussing the case and expected results with the patient and explaining the treatment plan to the patient.

After selecting nonprepared ceramic veneers as a treatment plan, gingival retraction was performed using a gingival retraction thread (SURE-CORD®, Sure Dent, Gyeonggi-do, South Korea). The final impression was taken using additional vinyl polysiloxane (CharmFlex®, DentKist, Gyeonggi-do, South Korea) in two stages with the Putty Wash Technique, using pre-made plastic trays (Tray Aways®, Keystone, Gibbstown, NJ). The gingival retraction thread was removed before the second stage.

This stage did not require anesthesia or abutment preparation, nor was a provisional restoration performed. The occlusal relationship was recorded in maximum intercuspation, and the veneer color was selected using the Vita Classic Color Guide (VITA classical A1-D4®, Vita, Zahnfabrik, Germany).

Ultrathin ceramic veneers were manufactured using lithium disilicate glass-ceramic (IPS e.max® Press, Ivoclar Vivadent, Schaan, Liechtenstein). The very thin thickness gradually decreased toward the gingival margin, recording a thickness ranging from 0.1 to 0.3 mm. The ceramic veneer was adequately trimmed to ensure ceramic thickness. Then, IPS E-max Ceram (IPS e.max® Ceram, Ivoclar Vivadent) building ceramic was applied and fired according to the manufacturer's instructions. IPS E-max Ceram Glaze (IPS e.max® Ceram Glaze, Ivoclar Vivadent) was applied to provide a glossy surface to the ceramic veneers.

Bonding of the ultrathin ceramic veneers

After completing the laboratory manufacturing stage of the ceramic veneers, they were sent from the laboratory for clinical try-in before bonding to ensure marginal adaptation and color harmony. Subsequently, the veneers were bonded to the nonprepared teeth using the following steps.

The internal surface of ultrathin ceramic veneers was cleaned with 99% alcohol, washed with water, and dried with air. The surface was then roughened with 9.5% hydrofluoric acid (Porcelain Etchant™, Bisco, Schaumburg, IL) for 20 seconds, washed with water, and dried with air until it had a chalky appearance, and silane (Porcelain Primer™, Bisco) was applied.

Abutment surfaces

Teeth were cleaned using fluoride-free pumice and rotary burs. The surface was roughened with 37% phosphoric acid (Tetric® N- Etch, Ivoclar Vivadent) for 30 seconds, washed with water, and dried with air until we had a chalky appearance. The bond (Tetric® N-Bond, Ivoclar Vivadent) was applied, followed by a light air stream and left without polymerization. Light-cured resin cement (Choice™ 2 Veneer Cement, Bisco) was applied to the internal surface of the ceramic veneer and bonded to the corresponding teeth. Adaptation and finger pressure were made, then cured using a light-curing device (LED.B Curing Light®, Woodpecker, Guilin, China) for five seconds. Excess bonding cement was removed using a dental probe and surgical blade.

Monitoring the clinical success of the fixed veneers

According to Walton's principles for evaluating clinical success and failure of fixed restorations, which the foreign direct investment approved in 2002, the fixed prosthodontic procedure was deemed "Complete success" if no restorative material was needed, whereas "Complete failure" was given to the case when restorative retreatment was required. The other terms used in this classification are given in Table 1 [22].

**Table 1:** Terms used in monitoring clinical success or failure of fixed prosthodontic restorations ^a^These definitions are based on Walton's principles [22]

Acronym	Definition^a^
CS	Complete success: direct examination of veneers showed no need for restorative retreatment
SU	Survival: restoration was not directly examined, but it was confirmed via telephone that no restorative retreatment was needed
UN	Unknown: the patient could not be followed up or contacted by phone
PF	Partial failure: restoration is acceptable functionally and esthetically despite minor complications, such as abfraction or fracture
CF	Complete failure: occurs when bonding failure, major fracture, or any issue requiring restorative retreatment

## Results

Baseline sample characteristics

The clinical study sample consisted of 60 ultrathin ceramic veneers manufactured from lithium disilicate glass-ceramic bonded to nonprepared upper teeth for six patients of both genders (three males and three females), as shown in Table 2. Each patient received 10 ceramic veneers for his/her upper 10 teeth (from the second premolar on the right-hand side to the second premolar on the left-hand side). The average age of the sample was 30.3 years, ranging from 23 to 44 years.

**Table 2:** Baseline sample characteristics of the included patients regarding age, gender, and the prepared teeth SD: standard deviation

Gender	Mean age (±SD)	The distribution of the teeth involved; n (%)		
		Anterior teeth (incisors and canines)	Premolars	All teeth (anterior teeth and premolars)
Males	31.0 (±11.3)	18 (60%)	12 (40%)	30 (100%)
Females	29.7 (±9.1)	18 (60%)	12 (40%)	30 (100%)
Both	30.3 (±9.2)	36 (60%)	24 (40%)	60 (100%)

The clinical success of the ceramic veneers

Table 3 presents the clinical success rate during the four assessment times.

**Table 3:** The results of assessing the clinical success rate of the fixed veneers at the four assessment times. CF: complete failure; PF: partial failure; UN: unknown; SU: survival; CS: complete success The definitions of these different categories are given in Table 1

Assessment time	Walton's classification of clinical success (n = 60)					
	CF	PF	UN	SU	CS	Total
After one month	0 (0%)	0 (0%)	0 (0%)	0 (0%)	60 (100%)	60
After three months	0 (0%)	0 (0%)	0 (0%)	0 (0%)	60 (100%)	60
After six months	0 (0%)	0 (0%)	0 (0%)	0 (0%)	60 (100%)	60
After one year	0 (0%)	0 (0%)	0 (0%)	0 (0%)	60 (100%)	60

The table indicates that all ceramic veneers were clinically successful regardless of the evaluation time. Therefore, there were no statistically significant differences in clinical success rates between the four assessment times (one month, three months, six months, and one year).

## Discussion

The term "clinical success of ceramic veneers" is primarily associated with clinical survival, which means the absence of significant functional, esthetic, and vitality issues during the follow-up period that would necessitate veneer replacement. This depends on the skill of the dentist and lab technician, as well as the patient's adherence to instructions [23]. The clinical success was evaluated according to Walton's principles, which assessed the success and failure of fixed prosthodontics. This classification has been adopted in most clinical studies, as it covers most of the cases encountered by the dentist during follow-up periods [22].

No failure cases were recorded in the study sample, with a clinical success rate of 100% for ultrathin ceramic veneers made from lithium disilicate glass-ceramic and bonded to nonprepared teeth during the 12-month follow-up period. This success can be attributed to the accurate diagnosis of the cases studied and the precise bonding of ceramic veneer, in addition to the advantages of nonprepared veneers, as completely bonding them to enamel is considered ideal in terms of bonding strength and minimal complications, along with patient compliance in maintaining oral hygiene.

The findings of this study are consistent with those of Smielak et al., who compared the clinical survival rate of feldspathic ceramic veneers using minimal preparation with the traditionally prepared veneers and found that the clinical survival rate for ceramic veneers with minimal or no preparation reached 100% over a relatively long follow-up period of nine years [1]. Additionally, the results of this study closely align with the results of Aslan's study, which conducted a retrospective study of the clinical survival rate of ceramic veneers made from lithium disilicate glass-ceramic using the heat-press technique, which reached 97.4% [24].

In contrast, the results of this study differ from those of Öztürk and Bolay's study, which evaluated the clinical performance of ceramic veneers bonded to enamel with and without exposure to dentin during preparation over a 24-month follow-up period, reporting a cumulative survival rate of 91.2%. This difference is attributed to overprepared cases associated with failures linked to bonding to exposed dentin [25].

Furthermore, the findings of this study differ from those of the systematic review of Layton and Clarke, who studied the clinical survival rate of ceramic veneers made from nonfeldspathic ceramic over a five-year follow-up period, reporting a clinical survival rate of 92.4%. This difference is due to the systematic review focusing on traditional ceramic veneers that require varying levels of tooth preparation [26]. Similarly, this study's results differ from the systematic review of Shebib et al., who studied the clinical survival rate of traditional ceramic veneers bonded to prepared teeth, reporting a survival rate of 90%. This difference arises from varying thicknesses of preparation for ceramic veneers and the weak mechanical properties of feldspathic ceramic compared to lithium disilicate glass [27].

Limitation of the current study

One limitation of the current work was the inability to evaluate cases with different types of malocclusion and poor oral hygiene. In addition, the current study's follow-up period is relatively short, and long-term follow-up studies are needed.

## Conclusions

Ultrathin ceramic veneers made from lithium disilicate glass-ceramic and bonded to nonprepared teeth represent a successful clinical and esthetic treatment option when evaluated using Walton's principles for assessing the success and failures of fixed prosthodontic procedures during the follow-up periods of the current research work at one month, three months, six months, and one year.
